# Quantification of EphA4 Turnover Rate and Subsequent Validation of Target Engagement for E2025, a Novel Anti‐EphA4 Antibody, in Human Neural Cells

**DOI:** 10.1002/alz70859_096834

**Published:** 2025-12-25

**Authors:** Takeo Kamakura, Kazuto Yamazaki, Akio Yamada, Kazuhiro Tahara, Kanta Horie, Eiji Inoue

**Affiliations:** ^1^ Eisai Co., Ltd, Tsukuba, Ibaraki Japan; ^2^ Eisai Co., Ltd., Kobe, Hyogo Japan; ^3^ Eisai Co., Ltd., Tsukuba, Ibaraki Japan; ^4^ Deep Human Biology Learning (DHBL), Eisai Inc., Nutley, NJ USA

## Abstract

**Background:**

EphA4 is a member of the ephrin receptor subfamily which is predominantly expressed in the brain. Activation of EphA4 is involved in neurodegeneration in several neurological diseases, including Alzheimer’s disease (AD) [1,2]. E2025 is a humanized anti‐EphA4 antibody that specifically binds to EphA4, leading to its deactivation. The concentration‐dependent changes in the turnover rate of EphA4 provide clear evidence of target engagement, which is critical for the development of E2025. In this study, we explored the turnover rate of EphA4 in human neural cells and the supernatants using Stable Isotope Labeling Kinetics (SILK) technology.

**Method:**

Endogenous cell proteins were labeled by adding ^13^C‐labeled leucine to the culture medium of human neural cells differentiated from HIP‐009 (human fetal hippocampal neural stem/progenitor cells). Subsequently, the medium was replaced with one containing unlabeled leucine together with E2025, and the supernatant and cells were collected at various time points. These samples were analyzed by high‐resolution LC/MS.

**Result:**

E2025 significantly increased the production rate of EphA4 in supernatants and promoted the degradation of EphA4 in cellsm. Furthermore, it was confirmed that E2025 promoted EphA4 turnover in a concentration‐dependent manner.

**Conclusion:**

SILK technology demonstrated that E2025 increased the turnover rate of EphA4 in a concentration‐dependent manner. Therefore, this method may be applicable for assessing the pharmacodynamic effects of E2025.

**References**

[1] Verma, M., Chopra, M. & Kumar, H. *Cell Mol Neurobiol* 43, 3375‐3391, (2023).

[2] Vargas, L. M., Cerpa, W., Munoz, F. J., Zanlungo, S. & Alvarez, A. R. *Biochim Biophys Acta Mol Basis Dis* 1864, 1148‐1159, (2018).

